# Individual slow wave events give rise to macroscopic fMRI signatures and drive the strength of the BOLD signal in human resting-state EEG-fMRI recordings

**DOI:** 10.1093/cercor/bhab516

**Published:** 2022-01-30

**Authors:** Merve Ilhan-Bayrakcı, Yuranny Cabral-Calderin, Til Ole Bergmann, Oliver Tüscher, Albrecht Stroh

**Affiliations:** Systemic Mechanisms of Resilience, Leibniz Institute for Resilience Research (LIR), 55122 Mainz, Germany; Neural and Environmental Rhythms, Max Planck Institute for Empirical Aesthetics, 60322 Frankfurt, Germany; Systemic Mechanisms of Resilience, Leibniz Institute for Resilience Research (LIR), 55122 Mainz, Germany; Neuroimaging Center (NIC), Focus Program Translational Neuroscience (FTN), University Medical Center of the Johannes Gutenberg University Mainz, 55131 Mainz, Germany; Systemic Mechanisms of Resilience, Leibniz Institute for Resilience Research (LIR), 55122 Mainz, Germany; Department of Psychiatry and Psychotherapy, University Medical Center of the Johannes Gutenberg University Mainz, 55131 Mainz, Germany; Systemic Mechanisms of Resilience, Leibniz Institute for Resilience Research (LIR), 55122 Mainz, Germany; Institute of Pathophysiology, University Medical Center of the Johannes Gutenberg University Mainz, 55131 Mainz, Germany

**Keywords:** brain states, EEG-fMRI, slow oscillations, slow wave events, thalamocortical networks

## Abstract

The slow wave state is a general state of quiescence interrupted by sudden bursts of activity or so-called slow wave events (SWEs). Recently, the relationship between SWEs and blood oxygen level–dependent (BOLD) functional magnetic resonance imaging (fMRI) signals was assessed in rodent models which revealed cortex-wide BOLD activation. However, it remains unclear which macroscopic signature corresponds to these specific neurophysiological events in the human brain. Therefore, we analyzed simultaneous electroencephalographic (EEG)-fMRI data during human non-REM sleep. SWEs individually detected in the EEG data were used as predictors in event-related fMRI analyses to examine the relationship between SWEs and fMRI signals. For all 10 subjects we identified significant changes in BOLD activity associated with SWEs covering substantial parts of the gray matter. As demonstrated in rodents, we observed a direct relation of a neurophysiological event to specific BOLD activation patterns. We found a correlation between the number of SWEs and the spatial extent of these BOLD activation patterns and discovered that the amplitude of the BOLD response strongly depends on the SWE amplitude. As altered SWE propagation has recently been found in neuropsychiatric diseases, it is critical to reveal the brain’s physiological slow wave state networks to potentially establish early imaging biomarkers for various diseases long before disease onset.

## Introduction

Slow wave activity (SWA) is a very well-characterized and intensively investigated multiscale activity state of the brain observable throughout various recording modalities, from multiunit activity in anesthetized mice (MUA) (Ruiz-Mejias et al. 2011) up to high density surface electroencephalography (EEG) in sleeping humans (Murphy et al. 2011; Siclari et al. 2018). During SWA, the membrane potentials of neurons alternate between depolarized Up and hyperpolarized Down states resulting in neuronal silence or a rather stereotypical bout of activity (Steriade et al. 1993). In the case of a synchronized transition from Down to Up state throughout a larger neuronal population, distinct slow wave events (SWEs) are detectable in the EEG. SWEs typically emerge when the cortex is functionally disconnected from the environment, and they are considered to represent the default activity pattern of the cortex and beyond (Sanchez-Vives and Mattia 2014). Importantly, the SWEs appear in a nonoscillatory fashion with the initiation and subsequent propagation of each SWE depending on the current local excitability (Sancristóbal et al. 2016). The SWEs occur with varying interevent intervals (IEI) during natural sleep (Riedner et al. 2007; Murphy et al. 2011) but tend to temporally cluster (Mölle et al. 2011); they can be induced by various anesthetic regimens (Seamari et al. 2007; Sakata and Harris 2009; Chen et al. 2013) and can even be recorded in cortical slabs (Timofeev et al. 2000). Although SWEs under different conditions might be phenomenologically similar, they might not share the underlying mechanisms in terms of SWE induction, particularly when comparing natural sleep with anesthesia (Akeju and Brown 2017; Reimann and Niendorf 2020). Moreover, SWEs can be found in diverse vertebrates, from birds to humans (Steriade et al. 2001; Riedner et al. 2007; Mascetti 2016) and are believed to be critical for memory consolidation (Diekelmann and Born 2010; Rasch and Born 2013; Tononi and Cirelli 2014; Timofeev and Chauvette 2017; Crunelli et al. 2018) and synaptic homeostasis (Tononi and Cirelli 2014) when occurring in natural sleep. The large-scale synchronization of SWEs as well as their local and widespread recruitment are highly sensitive to local and global excitability changes (Sancristóbal et al. 2016) and are also highly affected by cholinergic neuromodulation (Steriade 2004; Eggermann et al. 2014; Nghiem et al. 2020). This renders SWEs as a unifying functional network event which may allow using SWEs to study the network pathophysiology of neuropsychiatric disorders (Sanchez-Vives and Mattia 2014).

SWEs are not stationary events but propagate as traveling waves of activity from an initiation point (Massimini et al. 2004; Menicucci et al. 2009; Murphy et al. 2009). Spontaneous activity within the cortex itself, mainly in layer 5 is capable of initiating SWEs, even after thalamo-cortical deafferentation (Sanchez-Vives and McCormick 2000; Timofeev et al. 2000; Stroh et al. 2013). Under physiological conditions like sleep, spontaneous SWEs are predominantly generated locally in medial prefrontal, orbitofrontal, and insular regions (Massimini et al. 2004; Menicucci et al. 2009; Murphy et al. 2009). The propagation of slow wave events is critically dependent on the local excitability, that is, the excitability state of a limited number of neurons, or a local ensemble. Early stages of neurodegenerative and neuroimmunological disorders (Busche et al. 2015; Arnoux et al. 2018; Ellwardt et al. 2018) are marked by ensembles of hyperactive neurons and seem to prevent the propagation of SWEs (Busche et al. 2015). Although at later stages of these disorders neurodegeneration and neural hypofunction occur, early on, presumably due to maladaptive processes, a plasticity-driven hyperexcitability predominates (Sperling et al. 2009; O'Brien et al. 2010; Quiroz et al. 2010). These early shifts toward hyperactivity are quite subtle and have not yet been identified in human brain-wide imaging data. It is unclear how the blood oxygen level–dependent (BOLD) functional magnetic resonance imaging (fMRI) signal is related to the occurrence of individual SWEs during natural sleep in healthy humans. Resolving distinct BOLD fMRI patterns of SWEs in sleeping healthy humans is a critical prerequisite for any study examining SWE alterations reflecting early network disturbances caused by pathological conditions in humans. In rats, we have pioneered the combination of task-free, resting-state fMRI measurements and optical calcium recordings of spontaneous neural activity under anesthesia. We created a regressor based on individual SWEs for an event-related fMRI analysis and observed a cortex-wide BOLD pattern directly related to SWEs (Schwalm et al. 2017). Very recently, we could demonstrate that individual SWEs themselves drive a distinct fMRI activity signature (Aedo-Jury et al. 2020). In humans, Dang-Vu and colleagues examined BOLD signal changes related to neural events during sleep, including SWEs (Dang-Vu et al. 2008). In a group-based analysis, they observed significant BOLD signal increases in the pontine tegmentum, midbrain, cerebellum, parahippocampal gyrus, inferior frontal gyrus, middle frontal gyrus, precuneus, and posterior cingulate cortex (Dang-Vu et al. 2008) but not in areas critically involved in SWE recruitment, in particular other neocortical areas and the thalamus. We decided to perform our analyses primarily at a single-subject, single-session level and focused on individually detected SWEs, thus allowing the capture of interindividual and intraindividual differences without sacrificing information to group-level averaging. This is, in part, motivated by our aim to observe early neural network changes in neuropsychiatric disorders on an individual level. It also enables us to assess whether the amplitude and number of SWEs are quantitatively related to BOLD signal changes with single-subject resolution. The BOLD signal is related to subthreshold and suprathreshold neuronal activity via mechanisms of neurovascular coupling (Logothetis et al. 2010). It is yet to be shown whether the amplitude and quantity of a rather uniform neurophysiological event, in this case SWEs, translate to a proportional BOLD signal change.

Here, we introduce a translational approach: Inspired by our recent findings in rodents, we implement an event-related design using a clearly defined neurophysiological event as a regressor in task-free, resting-state fMRI recordings. We analyzed a combined EEG-fMRI dataset of 10 healthy human subjects (Bergmann et al. 2012), comprising two sessions of undisturbed night sleep per subject, and present single-subject, single-session brain activity patterns related to individual SWEs.

## Methods and Materials

### Origin and Characteristics of Source Data

The data used for this study are a subset of a previously published sleep study (Bergmann et al. 2012) that examined sleep spindle-related reactivation of category-specific cortical regions after learning face–scene associations.

These EEG-fMRI data were acquired by Til Ole Bergmann as part of the Project A6 “Neocortical processing modes of the sleeping brain as a neuronal substrate for memory consolidation in humans” (principal investigators: Hartwig R. Siebner, Lisa Marshall, Matthias Mölle) as part of the Collaborative Research Center SFB 654 “Plasticity & Sleep” at the Department of Neuroradiology, Neurozentrum, Christian-Albrechts University of Kiel, Germany.

The participants had no history of neurological or psychiatric disease and were not on medications. The experimental procedures were approved by the Ethics Committee of the University of Kiel, and all participants gave written informed consent prior to participation. For detailed information on the study protocol, please see Bergmann et al. (2012). A 32-channel EEG was acquired with an MR-compatible EEG cap (BrainCap MR, Easy-Cap, Munich, Germany) according to the 10–20 system. FCz was used as the reference electrode and the ground electrode was located at approximately 1 cm below the Oz position. Skin–electrode impedances were maintained below 5 kΩ throughout the recording (+5 kΩ safety resistors). Placing additional electrodes below and above the right eye, the outer canthi, the chin, and on the backbone allowed obtaining bipolar recordings of vertical and horizontal electrooculogram, electromyogram, and electrocardiogram. The data were recorded using BrainAmp MR plus DC and bipolar BrainAmp ExG MR amplifiers and the BrainVision Recorder V.1.10 software (BrainProducts, Munich, Germany) with a resolution of 0.5 μV/bit at 5 kHz and filtered between 0.016 and 250 Hz. The fMRI data were acquired on a 3 Tesla MR scanner (Philips Achieva; Philips Medical Systems, Best, The Netherlands). High-resolution T1-weighted anatomical images were collected using a standard MPRAGE sequence (TR = 7.7 ms, TE = 3.6 ms, flip angle = 8°, 170 sagittal slices, 1 × 1 × 1 mm voxel size, field of view = 224 × 224 mm). For sleep-fMRI, echo planar imaging (EPI) sequence was used (TR = 2240 ms, TE = 30 ms, flip angle = 90°, FOV = 216 × 216 mm, 38 transversal slices, slice thickness = 3 mm, gap = 10%, in plane voxel size = 3.38 × 3.38 mm, continuous bottom-up slice acquisition order).

To maintain a homogenous data structure throughout the sample, we studied the amount of time subjects spent in wakefulness (*M* ± SEM, 17.4 ± 4.2 min), in sleep stage N1 (*M* ± SEM, 2.4 ± 0.4 min), N2 (*M* ± SEM, 50.0 + 4 min), and N3 (*M* ± SEM, 38.6 + 3 min) (Supplementary Fig. 1). From the data of 11 participants at hand, we excluded one participant including their two sessions due to strong deviations in the amount of time spent in wakefulness (*M* = 67.1 min) and in sleep stage N2 (*M* = 9.6 min) from the sample mean. Ultimately, we examined data from 10 healthy, right-handed participants with two distinct sessions of night sleep per individual with a duration ranging from 53.8 to 149.2 min (*M* ± SEM, 107 ± 5.1 min).

### Data Analysis

#### E‌EG Preprocessing

The EEG data were preprocessed by MATLAB 9.6.0 (R2019a) (The Mathworks Inc.) and the EEGLAB v2019.0 toolbox (Delorme and Makeig 2004). Firstly, scanner gradient artifacts were removed using the “realignment parameter-informed” algorithm implemented in the Bergen toolbox in EEGLAB (Moosmann et al. 2009). This procedure is based on the moving average algorithm (Allen et al. 2000) and additionally takes head movement parameters from the fMRI signal into account to improve the estimation of artifact templates. Then, those artifact templates are subtracted from the distorted EEG signal. Next, EEG data are low-pass filtered at 200 Hz, downsampled to 200 Hz, and re-referenced to linked mastoids (i.e., TP9 and TP10). The EEGLAB plug-in FMRIB version 1.2 (Niazy et al. 2005) was used to remove electrocardiographic artifacts. To form the artifact template, we chose the optimal basis set (OBS) method and defined the number of principal components to be used as three. Finally, an independent component analysis (ICA) was performed to remove ocular, muscular, and the remaining electrocardiographic artifacts.

#### SWE Detection

SWEs were detected using a slow wave detection toolbox previously published by Mensen et al. (2016). The toolbox is based on an algorithm calculating the negative envelope of all electrodes. This approach can be imagined as creating a butterfly plot by overlaying all electrodes and then tracing the negative contour (Mensen et al. 2016). First, the negative-going signal envelope is calculated by selecting and averaging the three most negative samples across a set of 30 electrodes. Next, the resulting negative signal envelope is band-pass filtered between 0.5 and 4 Hz and baseline corrected (zero mean-centered). Finally, SWE is detected on the negative signal envelope by applying a validated procedure based on the detection of negative half-waves (Mölle et al. 2002; Massimini et al. 2004; Riedner et al. 2007; Nir et al. 2011; Siclari et al. 2014; Mensen et al. 2016; Bernardi et al. 2018; Siclari et al. 2018; Avvenuti et al. 2020; Betta et al. 2021). Here, negative half-waves with a duration of 250–1000 ms and passing the minimum amplitude threshold of 60 μV were considered SWEs. The onsets (timing of the first zero-crossing) and durations (from positive-to-negative zero-crossing to negative-to-positive zero-crossing) of each individually detected SWE were extracted to construct SWE vectors including the precise timing of the events. These event arrays were then used to analyze the simultaneously recorded resting-state fMRI data. For additional analyses, the peak amplitude (absolute value of the negative peak of the wave in the negative signal envelope in μV), the peak-to-peak amplitude (absolute value from the negative to positive peak of the wave in the negative signal envelope in μV), and the globality index (percentage of channels involved in each SWE) of each SWE were extracted.

The maximum propagation speed (m/s) of individual SWEs was determined by dividing the longest distance traveled by the maximum delay.

#### fMRI Preprocessing

The fMRI data were preprocessed and analyzed using statistical parametric mapping (SPM12, Wellcome Trust Centre for Neuroimaging, London, UK, http://www.fil.ion.ucl.ac.uk/) running on MATLAB 2019a (MathWorks, Natick, Massachusetts, USA). Due to equilibrium effects, the first four volumes of each scan were discarded. EPI volumes were slice-time corrected (with reference to the first slice of each volume) and realigned to the mean image (fourth order b-spline interpolation). Then, individual T1-weighted images were coregistered to the mean EPI, segmented, and transformed to Montreal Neurological Institute (MNI) space based on SPM’s tissue probability maps. Functional data were then normalized with the same spatial transformation and smoothed using a 6 mm full-width at half-maximum Gaussian smoothing kernel.

#### Event-Related fMRI Analyses

General linear models (GLM) were modeled comprising the SWE vector convolved with the canonical hemodynamic response function (HRF) as well as its derivatives in time and dispersion, six movement parameters (translations in *x*, *y*, and *z* directions and rotations around *x*, *y*, and *z* axes) and a constant term. We constructed an individual GLM for each subject and session as SWEs are highly variable with respect to the time of appearance and duration. To assess the BOLD response patterns associated with SWEs, we examined the main effect of SWEs relative to the implicit baseline. For that, we employed *F*-contrasts to draw inferences about the BOLD responses captured by the canonical HRF and the additional variability captured by its derivatives. Using *F*-contrasts allowed us to examine the overall BOLD signal changes related to SWEs. Results were considered significant for *P*_uncorr_ < 0.001 with a cluster extent threshold of 50 voxels at the voxel level and for *P*_FWEcorr_ < 0.05 at the cluster level. For further analyses, we calculated mean *F*-values for each individual BOLD response pattern comprising the *F*-values of all its voxels showing significant BOLD signal changes. To examine significant BOLD activity changes upon SWE occurrence in the cortex and thalamus separately, we generated ROI masks with WFU PickAtlas (Maldjian et al. 2003) and extracted the number of activated voxels and mean *F*-values within those regions.

The specificity of the SWE-related BOLD responses was tested by temporally mirroring the SWE vectors within the same recording sessions. The mirrored vectors contained the same number of events, the same distribution of event durations, and interevent intervals and only differed in the event onset. The mirrored SWE vectors were convolved with the canonical HRF and its temporal and dispersion derivative for event-related fMRI analyses.

#### Correlational Analyses

For correlational analyses, the number of SWEs and the mean SWE peak amplitude were determined for each subject and session. Both parameters were then correlated either with the number of activated voxels or the mean *F-*value derived from the results of the event-related fMRI analyses for the total brain, cortex, and thalamus, respectively.

As an additional parameter to use in correlational analyses, the percentage of high amplitude SWEs detected in each single session was defined. SWEs with a peak amplitude ≥124 μV (equals 1 SD above the mean amplitude across all sessions) were classified as “high-amplitude SWEs.” The number of SWEs surpassing this threshold (≥124 μV) were extracted and divided by the number of total SWEs detected in the individual sessions, and their extent of occurrence was described in percentages. This parameter was also then correlated to the number of activated voxels or the mean *F-*value derived from the results of the event-related fMRI analyses for the total brain, cortex, and thalamus, respectively (Supplementary Figs 2 and 3). Furthermore, the threshold for high-amplitude SWEs was varied to examine the consistency of the correlation indices. The variations included the following thresholds for SWEs to be considered a high-amplitude SWE: ≥100 μV, ≥140 μV and ≥160 μV (Supplementary Figs 2 and 3). Bivariate correlation analyses were performed on MATLAB 9.6.0 (R2019a) (The Mathworks Inc.). Results were considered significant for *P* < 0.05.

#### Examination of Interevent Intervals

To calculate the duration of intervals between two consecutive SWEs, here called interevent intervals (IEIs), the offset time of a SWE was subtracted from the onset time of a successive SWE. To produce the box-whisker plot, the IEIs of all SWEs detected throughout the whole sample were included (Fig. 2*B*). For the dot plot on the other hand, the individual median IEIs for each session were computed (Fig. 2*C*). Values outside 1.5 times the interquartile range (IQR) above the upper quartile (Q3) and below the lower quartile (Q1) were considered outliers and are not shown in the box-whisker plot and the dot plot depicted in Figure 2.

## Results

### Pipeline for Event-Related Analyses in Resting-State EEG-fMRI Recordings

**Figure 1:**
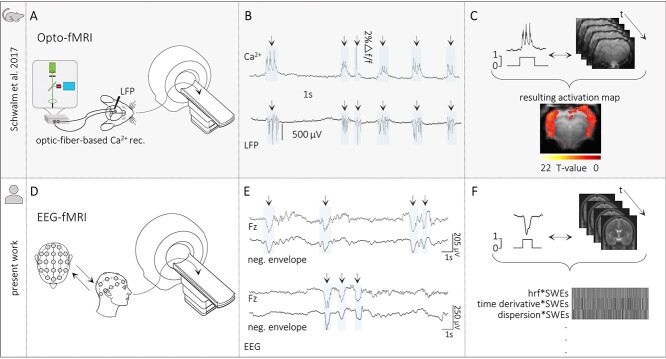
Translational approach for SWE-related fMRI analyses in humans. (*A*) Scheme of opto-fMRI setup enabling simultaneous fiber-based calcium and fMRI recordings in anesthetized rodents. (*B*) Traces of simultaneous optical calcium and local field potential (LFP) recordings implying a correlation of slow calcium waves with electrically recorded SWEs under isoflurane anesthesia. (*C*) Upper part: Scheme of the data analysis procedure. Slow calcium wave events were converted to SWE vectors that were included in subsequent event-related fMRI analyses. Lower part: BOLD activation pattern upon event-related fMRI analysis in the rat brain. Panels *A*, *B*, and *C* are adapted by courtesy of Schwalm et al. (2017). (*D*) Scheme of simultaneous EEG-fMRI setup in sleeping humans. (*E*) Excerpts of EEG traces to illustrate successful detection of individual SWEs in the present EEG data. (*F*) Upper part: Scheme of data analysis procedure in the present study—Analogous to animal data analysis procedure. Individually detected SWEs were converted to SWE vectors including the precise timing of the events. Lower part: Depiction of an exemplary fMRI design matrix containing among others the SWE vector convolved with the canonical HRF and its temporal and dispersion derivative.

Firstly, we employed optic fiber–based calcium recordings alongside resting-state fMRI recordings to identify the brain-wide BOLD-signal correlate of SWE in anesthetized rats (Fig. 1*A–C*). We have already demonstrated (Stroh et al. 2013; Schwalm et al. 2017) that electrical recordings of population activity and optical population recordings of action potential–related intracellular calcium elevations capture identical SWEs (Fig. 1*B*). The individually detected slow calcium waves were used as regressors in event-related fMRI analyses (Fig. 1*C*; Friston et al. 1998). Using event-related fMRI analyses, we previously observed a cortex-wide BOLD signal correlated with individually detected slow calcium waves (Schwalm et al. 2017; Aedo-Jury et al. 2020).

For the translation of the abovementioned approach of using neurophysiological events as regressors in event-related fMRI analyses, in the present study, we analyzed human sleep EEG-fMRI data as SWEs typically emerge during natural sleep. (Fig. 1*D–F*). We examined data from 10 healthy participants with two distinct sessions of night sleep per individual. Data had been acquired and published in a previous study by Bergmann et al. (2012).

In analogy to the slow calcium waves optically recorded in anesthetized rodents, 32-channel EEG recordings were used to identify individual SWEs based on their EEG signature (Fig. 1*E*). SWEs were detected using a published slow wave detection toolbox (Mensen et al. 2016). For the detection of SWEs, first, a canonical signal, that is, the negative-going signal envelope across all EEG channels was calculated. Next, SWEs were detected in the negative signal envelope by the application of a procedure based on the detection of negative half-waves (Mölle et al. 2002; Riedner et al. 2007). Negative half-waves having a duration between 250 and 1000 ms and passing the minimum amplitude threshold of 60 μV were considered SWEs. In general, the procedure to identify SWEs based on the detection of negative half-waves can be applied to a variety of canonical signals. A canonical signal can be obtained, for example, by calculating the mean activity over a single circular region of the electrode array. Instead, multiple canonical waves can be computed by taking the mean activity of, for example, four regions equidistant around the center as previously applied by Massimini et al. (2004). Regional signal averaging approaches have some disadvantages, such as the canonical wave will not take local SWEs outside the specified regions into account. Thus, they cannot be detected. In contrast, regions specified too large might result in a mean activity which is no longer representative for that area. Again, SWEs could be missed. Choosing the negative signal envelope as a canonical signal, as done in the present work, solves the abovementioned potential issues and holds several advantages. The most negative samples can originate from any channel in the data and are not limited to a specific region. In addition, one canonical signal is representative of the whole dataset. Furthermore, having no regional restrictions allows the identification of both local and widespread SWEs with a precise time reference across all channels (Mensen et al. 2016).

**Figure 2:**
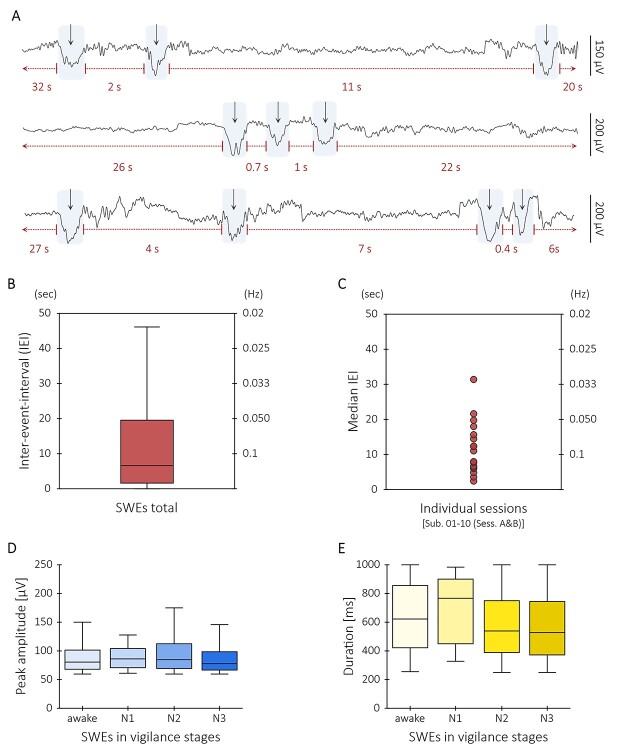
SWEs are characterized by varying IEIs both on intra- and interindividual level. (*A*) Three different exemplary EEG data segments of a single subject illustrate the variety of IEIs of SWEs. The rows show epochs (≈ 18 s) of artifact-corrected EEG signal of channel Fz. (*B*) Box-whisker plot shows the distribution of IEIs of all SWEs detected in this study. (*C*) Depiction of median IEIs of *n* = 17 individual sessions. (*D*) Box-whisker plots illustrate the distribution of peak amplitudes (μV) of all SWEs occurring in wakefulness, in sleep stage N1, in sleep stage N2, and in sleep stage N3, respectively. Mann–Whitney U tests indicated that peak amplitudes of awake SWEs (Mdn = 81.2 μV) did not significantly differ from the peak amplitudes of N1 SWEs (Mdn = 81.2 μV) (*U* = 2093, *P* = 0.85), of N2 SWEs (Mdn = 84.6 μV) (*U* = 174 773, *P* = 0.19), and N3 SWEs (Mdn = 78.1 μV) (*U* = 349 908, *P* = 0.08). Furthermore, peak amplitudes of N1 SWEs did not significantly differ from the peak amplitudes of N2 SWEs (*U* = 20 037*, P* = 0.74) and N3 SWEs (*U* = 32 900*, P* = 0.4). However, the peak amplitudes of N2 SWEs and N3 SWEs significantly differed from each other (*U* = 2 753 639*, P* < 0.001). (*E*) Box-whisker plots show the distribution of durations (ms) of all SWEs appearing in wakefulness, in sleep stage N1, in sleep stage N2, and in sleep stage N3, respectively. Although the durations of SWEs occurring in wakefulness (Mdn = 620 ms) did not significantly differ from the durations of N1 SWEs (Mdn = 765 ms) (*U* = 1870*, P* = 0.85), they were significantly longer than the durations of N2 SWEs (Mdn = 540 ms) (*U* = 157 876*, P* < 0.001) and N3 SWEs (Mdn = 525 ms) (*U* = 269 824, *P* < 0.001). Likewise, the durations of N1 SWEs were significantly longer than the durations of N2 SWEs (*U* = 14 927, *P* = 0.02) and N3 SWEs (*U* = 25 404, *P* = 0.01). There was no significant difference between the durations of N2 and N3 SWEs (*U* = 3 084 885, *P =* 0.09).

The traces of the individual electrodes displayed temporal dynamics typical for slow wave activity: SWEs did not occur at a fixed frequency as, for example, delta oscillations (Walter 1937) but in rather temporally broad interevent intervals (Fig. 2*A–C*, Supplementary Fig. 4). Here, the interquartile range of the interevent intervals of all SWEs was from 1.5 to 17 s (0.7–0.06 Hz), well below the frequency of delta oscillations which rather appear in the range between 1 and 4 Hz (Steriade 2006).

SWEs are not limited to distinct sleep stages and can even occur albeit locally in the awake condition (Vyazovskiy et al. 2011). We did not differentiate our analysis based on these stages but included all vigilance stages. Even in rather brief periods of wakefulness, we could detect SWEs. Characteristics like event duration (ms) and peak amplitude (μV) of SWEs occurring throughout different vigilance stages were in similar ranges (Fig. 2*D*,*E*, Supplementary Fig. 5). The peak amplitudes of SWEs occurring in sleep stage N2 were slightly higher than those of N3 SWEs and the durations of awake and N1 SWEs were longer than the durations of N2 and N3 SWEs.

The simultaneous recording of SWE dynamics by 32 EEG electrodes allowed for the identification of temporal delays with regard to the onset of the individual SWEs. We found a subset of SWE temporal delays with SWE propagation (Supplementary Fig. 6). The median propagation speed of 2.4 m/s is well within the range of earlier studies (Massimini et al. 2004; Avvenuti et al. 2020).

For the analysis of the simultaneously recorded task-free fMRI recordings, the onset and duration of each individually detected SWE were extracted and vectors including the precise timing of the SWEs were constructed.

### Macroscopic BOLD Signature of SWEs

**Figure 3:**
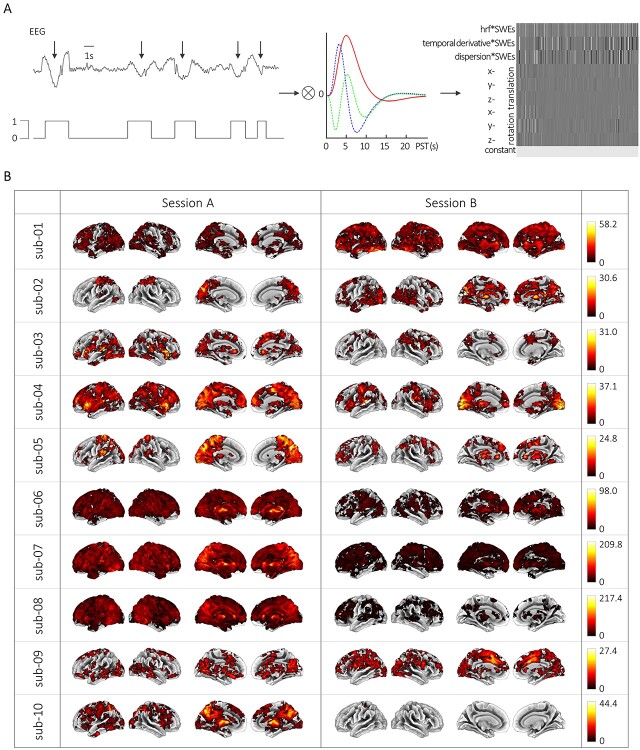
SWE-related hemodynamic changes at the single-subject, single-session level. (*A*) Scheme of the fMRI data analysis procedure. From left to right: Individually detected SWEs were converted to SWE vectors including the precise timing of the events. These SWE vectors were convolved with the canonical HRF and its temporal and dispersion derivative for event-related fMRI analyses. An exemplary design matrix shows the individual GLM which contains the SWE vector convolved with the canonical HRF and its derivatives in time and dispersion, six movement parameters, and a constant term. (*B*) BOLD activation maps showing significant changes upon SWE appearance (voxel-level: clusters >50 voxels at *P*_uncorr_ < 0.001; cluster-level: *P*_FWEcorr_ < 0.05) for sessions A and B of all subjects, respectively. Color bars indicate *F*-values. Of note, intrasubject variability can be appreciated comparing between sessions, for example, sub-04 session A versus sub-04 session B*.*

Based on the SWE vectors, we modeled individual GLMs (see Figs 1*F* and 3*A*). For that, we convolved the SWE vector with the canonical HRF and its derivatives in time and dispersion and included six movement parameters and a constant term. We examined the main effect of SWEs relative to the baseline to see if there is a specific BOLD signature in response to SWEs.

Overall, 5459 (*M* ± SEM, 273 ± 53.1) SWEs per session per participant were identified in the present analysis. Employing the aforementioned approach using a regression vector derived from these SWEs resulted in distinct BOLD activation maps in 20 out of 20 recording sessions (sub-01 to sub-10, sessions A and B, Fig. 3*B* and Table 1). In seven sessions, we found large, spatially connected significant clusters exceeding 20 000 voxels (sub-01-B, sub-04-A, sub-06-A, sub-06-B, sub-07-A, sub-07-B, and sub-08-A). These connected clusters covered large sections of the cortex typically including the occipital, parietal, temporal, and frontal lobes. These spatial activation patterns are reminiscent of cortex-wide activations in rodent models (Schwalm et al. 2017; Aedo-Jury et al. 2020). In 12 further sessions, the number of voxels showing significant BOLD signal changes upon SWE occurrence ranged between 4161 and 17 519 voxels (sub-01-A, sub-02-A, sub-02-B, sub-03-A, sub-03-B, sub-04-B, sub-05-A, sub-05-B, sub-08-B, sub-09-A, sub-09-B, and sub-10-A). However, for one session, a single localized significant cluster showing BOLD signal changes in 62 voxels was identified (sub-10-B).

**Table 1:** BOLD signal changes in SWE-related brain activity

	Session A						Session B					
	Cluster level		Peak level				Cluster level		Peak level			
ID	*p*_FWE-corr_	*k*_E_	*x*	*y*	*z*	*F-*value	*p*_FWE-corr_	*k*_E_	*x*	*y*	*z*	*F-*value
sub-01	<0.001	14 391	54	−67	29	24.69	<0.001	34 236	−6	−76	−34	58.20
sub-02	<0.001	3838	−9	−79	38	25.82	<0.001	12 198	9	−79	−22	30.60
sub-03	<0.001	12 273	−42	−31	11	30.96	<0.001	1100	−39	−73	−37	17.14
sub-04	<0.001	23 119	33	20	−4	33.68	<0.001	10 267	9	−91	−10	37.08
sub-05	<0.001	8778	9	−70	35	24.78	<0.001	1176	3	−79	−22	21.60
sub-06	<0.001	44 500	−33	−67	−28	98.04	<0.001	20 432	6	−73	−25	51.65
sub-07	<0.001	53 061	0	2	2	209.83	<0.001	29 525	−51	−61	−34	50.21
sub-08	<0.001	49 391	−27	−61	−28	217.39	<0.001	8466	−30	−61	−28	45.53
sub-09	<0.001	8261	−45	−82	8	25.67	<0.001	13 309	30	−40	−31	27.41
sub-10	<0.001	17 432	0	−70	−22	44.40	<0.001	62	−33	−40	62	8.98

*Notes*: The table shows MNI coordinates (*x*, *y*, *z*) and *F*-values for clusters (*k*_E_) holding peak BOLD signal changes for each individual subject and session significant at *P*_uncorr_ < 0.001 (cluster size >50 voxels) at voxel level and *P*_FWEcorr_ < 0.05 at cluster level.

The approach to employ neurophysiological events as regressors as in classical event-related fMRI analyses enabled us to identify differential BOLD signatures of SWEs both in cortical and subcortical structures.

Most prominently, the strongest BOLD signal changes within the individual sessions were revealed in the cerebellum (sub-01-B, sub-02-B, sub-03-B, sub-05-B, sub-06-A, sub-06-B, sub-07-B, sub-08-A, sub-08-B, sub-09-B, and sub-10-A). Furthermore, areas with peak BOLD signal changes included the parietal lobe (sub-01-A, sub-02-A, sub-05-A, and sub-10-B) and the occipital lobe (sub-04-B and sub-09-A). For three further sessions, the peak BOLD signal changes were in either the temporal lobe (sub-03-A), the frontal lobe (sub-04-A), or the thalamus (sub-07-A) (Table 1).

Notably, upon SWE appearance significant BOLD signal changes in the thalamus were found in 17 recording sessions (Table 2). The occurrence of BOLD signal changes in relation to SWEs in the thalamus are in line with earlier studies demonstrating a strong involvement of the thalamic circuit in SWE dynamics (Steriade et al. 1993; Timofeev et al. 2001; Stroh et al. 2013; Sheroziya and Timofeev 2014).

**Table 2:** Thalamic BOLD signal changes upon SWE occurrence.

	Session A								Session B							
	Left thalamus				Right thalamus				Left thalamus				Right thalamus			
ID	*x*	*y*	*z*	Voxels	*x*	*y*	*z*	Voxels	*x*	*y*	*z*	Voxels	*x*	*y*	*z*	Voxels
sub-01	-	-	-	-	3	−19	11	63	−6	−16	11	227	12	−10	2	285
sub-02	-	-	-	-	-	-	-	-	3	−16	8	83	−3	−19	8	62
sub-03	−3	−19	8	57	-	-	-	-	-	-	-	-	-	-	-	-
sub-04	−15	−19	11	159	9	−19	5	134	−6	−19	5	190	6	−19	2	110
sub-05	9	−19	2	68	−9	−19	11	103	−6	−13	8	66	6	−10	5	94
sub-06	9	−31	5	305	−3	−16	8	296	9	−31	8	234	−3	−19	11	225
sub-07	3	−22	8	299	−3	−16	11	287	−6	−4	5	164	3	−22	8	190
sub-08	3	−4	5	286	−3	−7	5	286	−3	−7	5	123	3	−7	5	88
sub-09	21	−34	2	106	−12	−25	11	59	-	-	-	-	6	−19	−1	51
sub-10	3	−10	11	201	−3	−16	8	202	-	-	-	-	-	-	-	-

*Notes*: The table provides the number of activated voxels within the right and left thalamus and MNI coordinates (*x*, *y*, *z*) for peak BOLD signal changes within these regions for each individual subject and session, respectively.

To further test for the specificity of the SWE vectors to the related BOLD responses, a control procedure was conducted. SWE vectors were temporally mirrored and resulted in no BOLD responses for 15 out of 20 recording sessions. For the remaining five sessions (sub-03-A, sub-04-B, sub-05-B, sub-06-B, and sub-10-B), there were random localized BOLD responses with *F*-values substantially differing from the responses obtained by the original SWE vectors (Supplementary Fig. 7).

### Amplitude and Number of SWEs Significantly Correlate to the Spatial Extent of the BOLD Patterns

**Figure 4:**
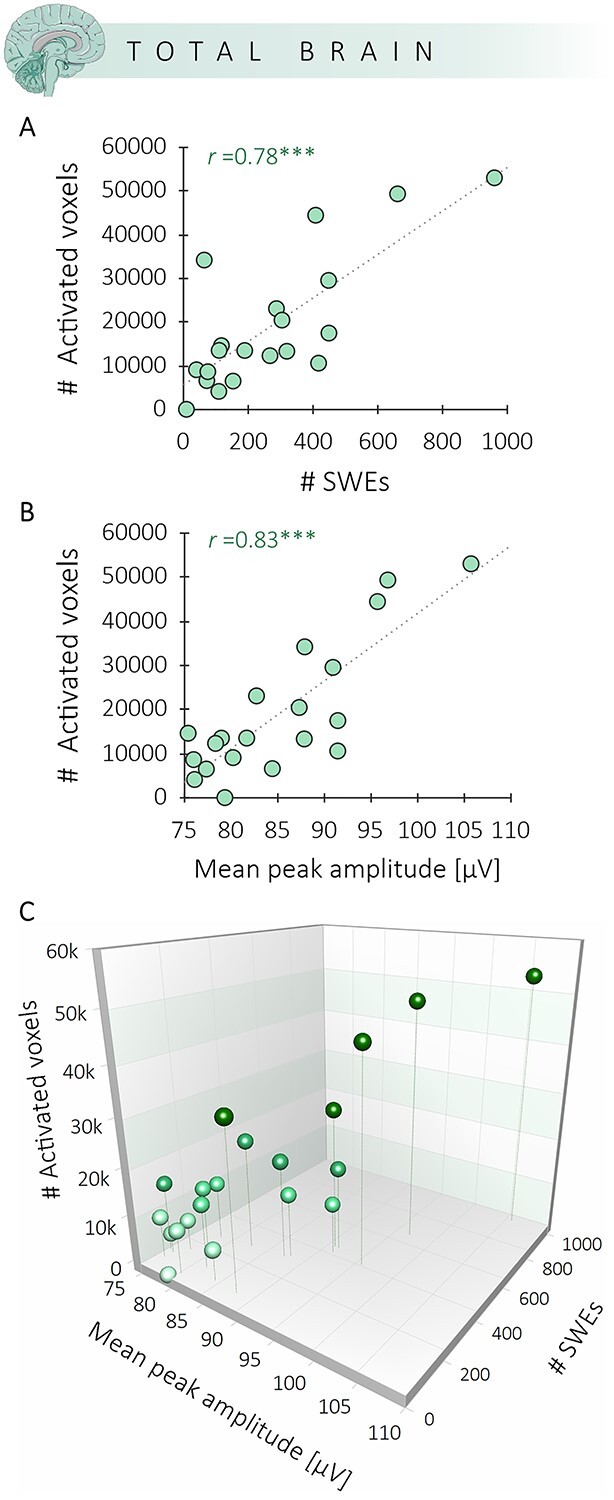
The spatial extent of BOLD signal changes significantly correlates with the number and amplitude of SWEs. (*A*) Correlation between the number of activated voxels upon SWE occurrence and the number of SWEs. (*B*) Correlation between the number of activated voxels upon SWE occurrence and the mean peak amplitude of the SWEs. (*C*) Panels A and B displayed in a 3D scatter plot. ^***^The correlations are significant at *P* < 0.001.

Although we could identify in individual subjects a widespread BOLD activation upon SWE occurrence, in other subjects and sessions we observed rather small clusters. The number of SWEs varied dramatically between sessions, ranging from 9 up to 960 SWEs (*M* ± SEM, 273 ± 53.1). We therefore asked whether the spatial extent of the BOLD responses depend on the number of SWEs. We indeed found a significant correlation between the number of activated voxels and the number of SWEs (*r* = 0.78, *P* < 0.001) (Fig. 4*A*,*C*). More SWEs lead to larger spatially connected clusters showing BOLD signal changes. Note that as SWEs are detected in the canonical wave, the number of SWEs is independent from the number of EEG electrodes involved in SWEs across a session. The correlation between number of SWEs and the number of activated voxels of SWE-related BOLD responses is therefore independent from the SWE globality. Additional analyses showed no significant correlation between mean globality indices and the number of activated voxels (*r* = 0.19, *P* = 0.43, Supplementary Fig. 8).

Furthermore, the average SWE peak amplitude across all detected SWEs within a session significantly correlated with the number of activated voxels (*r* = 0.83, *P* < 0.001), which might point to the strength of the local recruitment of the respective neuronal ensemble to the SWE (Fig. 4*B*,*C*). In particular, the high amplitude SWEs (peak amplitude ≥124 μV) seem to drive the spatial extent of the BOLD activation patterns (Supplementary Figs 2 and 9).

**Figure 5:**
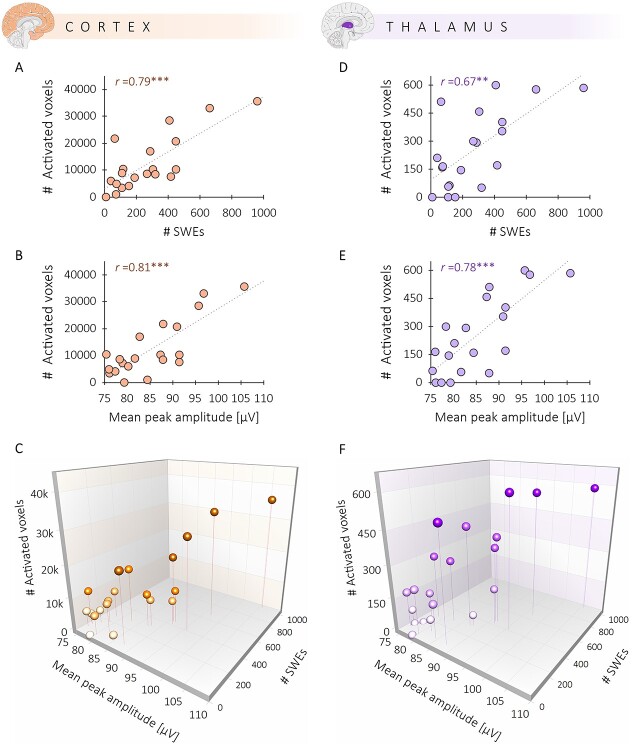
Correlation between SWEs and BOLD is present in two regions critical for SWE initiation and recruitment. (*A*) Correlation between the number of activated voxels within the cortex and the number SWEs. (*B*) Relation between the number of activated cortical voxels and the mean peak amplitude of SWEs. (*C*) A 3D scatter plot illustrates both correlations for cortical activation. (*D*) Correlation between the number of activated voxels within the thalamus and the number of SWEs. (*E*) Relation between the number of activated thalamic voxels and the amplitude of SWEs. (*F*) Both correlations for thalamic activation displayed in a 3D scatter plot. ^***^The correlations are significant at *P* < 0.001. ^**^The correlation is significant at *P* < 0.01.

These findings strengthen the notion of a specific spatial BOLD signature of SWEs. As the cortex and the thalamus are critical for the initiation and recruitment of SWEs (Crunelli and Hughes 2010; Stroh et al. 2013; Crunelli et al. 2015), we examined cortical and thalamic BOLD signal changes upon SWE occurrence separately. The correlation between the number of activated voxels within those regions and the number of SWEs remained highly significant for the cortex (*r* = 0.79, *P* < 0.001) (Fig. 5*A*,*C*) as well as the thalamus (*r* = 0.67, *P* < 0.01) (Fig. 5*D*,*F*). Similarly, a higher average peak amplitude of SWEs leads to more activated voxels in the cortex (*r* = 0.81, *P* < 0.001) (Fig. 5*B*,*C*) and the thalamus (*r* = 0.78, *P* < 0.001) (Fig. 5*E*,*F*). Furthermore, the spatial extent of a cortical activation significantly correlates to the spatial extent of the thalamic activation (*r* = 0.85, *P* < 0.001) (Supplementary Table 1). The same correlation could be found for the amplitude, that is, the mean *F*-value of the cortical and thalamic BOLD response (*r* = 0.92, *P* < 0.001) (Supplementary Table 1).

### Direct Relation Between the Amplitude of a Neuronal Event and the Amplitude of the Related BOLD Response

**Figure 6:**
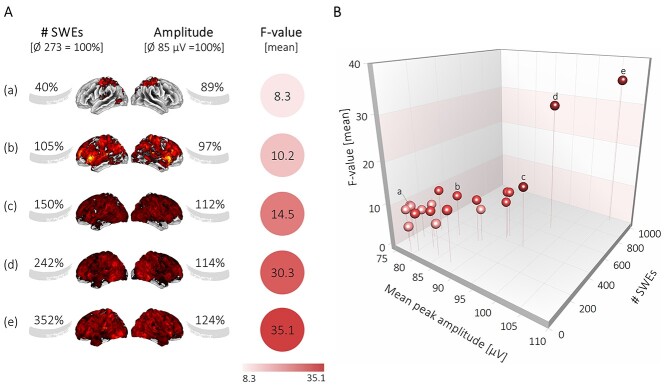
Amplitude of the SWEs drives the amplitude of the BOLD signal. (*A)* Illustration shows that higher numbers of SWEs and higher mean peak amplitudes of SWEs give rise to stronger mean *F*-values in the BOLD signal. (*B*) 3D scatter plot displays the correlation between mean *F*-values and the mean peak amplitude of SWEs (*r* = 0.79*, P* < 0.001) and between mean *F*-values and the number of SWEs (*r* = 0.85*, P* < 0.001). Individual dots marked with letters correspond to the examples in panel A.

The identification of singular, but uniform, SWEs using a negative envelope detection method and their fMRI modeling as individual events allows the assessment of the potential coupling of the amplitude of the SWEs and the amplitude of the BOLD signal. Notably, we revealed a significant correlation between the average amplitude of the SWEs and the normalized BOLD response, that is, the mean *F*-value (*r* = 0.79, *P* < 0.001) (Fig. 6). Subjects with a higher-than-average SWE amplitude and higher-than-average number of SWEs displayed a significantly stronger BOLD response. Here again, especially the high amplitude SWEs (peak amplitude ≥124 μV) had a substantial effect on the magnitude of the *F*-values (Supplementary Figs 3 and 9). Importantly, we found a linear correlation between the variables SWE amplitude, SWE number, and the amplitude of the BOLD signal (Fig. 6).

## Discussion

Neurophysiologically well-defined SWEs can be found in mice and humans, and their main features seem to be highly preserved across species. SWEs may function as initiators of local and brain-wide neural synchronization allowing information transfer between distant brain regions and likely being involved in processes like memory consolidation (Tononi and Cirelli 2014; Timofeev and Chauvette 2017; Crunelli et al. 2018) and synaptic homeostasis (Tononi and Cirelli 2014). SWE occurrence and propagation are highly susceptible to excitability changes and states of their network (Huber et al. 2004; Huber et al. 2006; Stroh et al. 2013; Busche et al. 2015; Sanchez-Vives et al. 2017). This susceptibility makes SWEs an ideal target for studying brain (dys-)function across species and across diseases. Even though the original characterization of slow oscillations by Mircea Steriade and collaborators date back to the early 1990s (Steriade et al. 1993), the potential of SWEs as a central marker of network state has not been fully exploited in human and translational research yet. SWEs occur over multiple scales, from single neurons—here termed Up-Down state transitions—to large-scale EEG—here termed slow oscillations. SWEs transgress individual fields of neuroscience, and therefore yield different termini for a unifying event. In particular, in the field of human neuroimaging, there had not been a clear focus on studying SWEs. Here, we started our analyses by thoroughly defining SWEs in combined EEG-fMRI measures in healthy human subjects. We did not spatially bias the SWE detection procedure by applying it on averaged region of interest (ROI) signals over predefined regions. Performing the SWE detection procedure on the negative signal envelope of all EEG channels allowed capturing SWEs appearing in any channel without being limited to a specific region. Furthermore, dispensing regional restrictions facilitates the identification of both local and widespread SWEs. Each SWE detected was used to construct a SWE vector to identify the brain-wide signature of SWEs in human neuroimaging data. Moreover, this approach allows for a post-hoc analysis of the specific signatures of local versus widespread SWEs.

### A Framework for Defining SWEs in EEG-fMRI Data

It is important to note, that in our view, the term “slow oscillations,” still widely used in the field, misrepresents key features of SWEs, chiefly the fact, that these events do not carry a fixed frequency and are not governed by a pacemaker. Each SWE is an event in its own right, depending on the buildup of local state of excitability, either due to spontaneous activity or due to afferent input (Crunelli and Hughes 2010; Crunelli et al. 2015; Pachitariu et al. 2015; Schwalm et al. 2017). We previously demonstrated that SWEs can be evoked by the stimulation of less than 50 neurons in layer 5 mouse cortex (Vyazovskiy et al. 2009; Stroh et al. 2013). Subcortical structures, mainly thalamic nuclei, are involved in SWE initiation (Crunelli and Hughes 2010; Stroh et al. 2013; Sheroziya and Timofeev 2014; Crunelli et al. 2015), but, again, not in the sense of a rigid pacemaker. This clearly separates the SWEs from true oscillations, such as delta oscillations. The hallmark of SWEs is their varying interevent intervals. Delta oscillations, on the other hand, can be easily identified in the FFT as distinct peaks, normally at around 1–4 Hz (Steriade 2006).

There are various approaches to detect SWEs in EEG data as there is neither a standard SWE detection method nor consensus on the detection parameters (Mensen et al. 2016). Picot et al. (2012) proposed a detection method for SWEs in EEG recordings by a matching pursuit algorithm using Gabor functions reproducing the main targeted waveform characteristics. Moreover, the “fMRI artifact rejection and sleep scoring” toolbox offers the option to detect SWEs in EEG recordings (Leclercq et al. 2011). The detection is performed on averaged ROI signals (i.e., over four averaged signals) and based on criteria such as amplitude, slope, and wave duration (Massimini et al. 2004). In our previous related work, we employed an algorithm put forward by Maria V. Sanchez-Vives’ group (Seamari et al. 2007) and adapted it for the identification of SWE-associated calcium waves (Schwalm et al. 2017; Aedo-Jury et al. 2020). The algorithm separates slow oscillatory activity and periods of network quiescence based on exponential moving average (EMA) filters. Here, we used the “swa-matlab” toolbox put forward by Giulio Tononi’s group as it holds numerous advantageous features and it is an attempt to standardize SWE detection for reliable comparisons of results across studies (Mensen et al. 2016). Of note, the SWE detection algorithm is not able to differentiate between SWEs and K-complexes. For that, simultaneous recordings of multiunit activity (MUA) and electrophysiology would be necessary as conducted in Cash et al. (2009). The open-source MATLAB-based toolbox (The Mathworks Inc) provides a user-friendly interface allowing the detection and analysis of a variety of properties of individual SWEs, such as amplitude, slopes, topographic location, and globality. In particular, the detection algorithm based on the calculation of the negative signal envelope is a highly suitable tool in the context of our research as it has enhanced sensitivity to detect widespread SWEs as opposed to, for example, the calculation of the mean activity of a specified region. Here, 95% of the SWEs detected were of widespread nature (>50% of electrodes recruited by a SWE). This feature is of high value for future examinations of early network dysregulations in slow wave activity in neurological disorders as particularly the widespread SWEs are impeded, for example, due to local ensembles of hyperactivity (Busche et al. 2015).

### Signature of SWE in Human Neuroimaging Data

Particularly for the long-standing question “to which extent resting-state fMRI signature corresponds to neurophysiological events or other physiological or non-physiological sources”, slowly fluctuating signals in EEG were correlated to fMRI BOLD contributing to our current understanding that at least a substantial component of the resting-state fMRI signal reflects changes in neuronal activity (Pan et al. 2013; Thompson et al. 2014). We focused our efforts in converting the task-free, resting-state fMRI data post hoc into an event-related fMRI design. We did not correlate these two signals, EEG on the one hand and fMRI on the other hand, but constructed regression vectors of the SWEs detected via the EEG to perform an event-related fMRI analysis. We pioneered this approach in rodents with simultaneous optic fiber–based identification of SWE-associated slow calcium waves and fMRI, back-to-back with Markus Rudin’s group (Schlegel et al. 2018). We did not attempt to identify all sources of the fMRI BOLD signal fluctuations but extracted the component which is directly related to the occurrence of individual SWEs. In this study, we took a decisive next step and implemented an analysis on the correlation between both the number of SWEs and the strength of the local recruitment, that is, the amplitude of SWEs. Particularly the SWE analysis toolbox used here enables a quantitative and robust measure of SWE amplitudes and SWE globality.

A seminal previous study in humans reported slow waves to be predominantly local in nature (Nir et al. 2011). The authors analyzed simultaneously recorded scalp EEG, intracerebral EEG, and unit firing in multiple brain regions of neurosurgical patients. This provided them with maximum high temporal but somewhat limited spatial resolution as intracerebral EEG and unit firings were almost exclusively recorded in the cortical and subcortical structures. Compared to Nir and colleagues, our simultaneous EEG-fMRI approach is surely limited by the comparably poor temporal resolution of the BOLD fMRI response. However, by selecting singular SWEs often being separated by seconds rather than continuous (rhythmic) slow wave activity (as in Nir et al. 2011), we may have picked different phenomena in the two studies. The selection of singular, temporally spaced SWEs also lessens the problem of the temporally ill-defined BOLD response as the temporal occurrence of the SWEs is in the range of the BOLD response and perfectly fits event-related fMRI designs. In addition, whole brain fMRI prevents the spatial bias which is inherent to intracerebral EEG and unit firing recordings and may therefore pick up more spatially distributed activity. However, high temporal relation of scalp EEG to intracerebral EEG and unit firing may for sure imply causal relation of intra- and extra-cerebral electrical activity whereas EEG-fMRI is by far more correlational. In sum, both studies provide rather complementary than contradictory results concerning the nature of sleep-related slow waves and SWEs, respectively.

At this point, it is important to make use of the synergistic information provided by simultaneous EEG-fMRI recordings. Although EEG exhibits a high temporal resolution it lacks the spatial precision to resolve subcortical structures. In contrast, fMRI mainly lacks temporal resolution but can provide valuable information on the spatial signature of SWEs and particularly on the involvement of subcortical structures, such as the thalamus.

### Neurophysiologically Defined SWEs Drive the Spatial Extent and Amplitude of the BOLD Signal

Deciphering the principles of neurovascular coupling represents a vivid field of research; it is very likely that multiple mechanisms are involved (Uhlirova et al. 2016; Iadecola 2017; Mishra 2017). Our current understanding is that the BOLD response is mainly driven by synaptic activity (Logothetis et al. 2010). However, quantitative interpretations of the amplitude of a BOLD signal to the underlying output, that is, spiking activity of a given region remains inconsistent. The SWE as measured via EEG reflects the population equivalent of the transition of neurons from Down to Up state. These transitions govern both subthreshold, synaptic activity, and spiking activity as both are drastically reduced in the Down state. While the individual contributions of sub- versus suprathreshold activity to the BOLD signal can still not be quantified, it is clear that the onset of a SWE at a given location of a network signifies a drastic change in local excitability (Bergmann et al. 2012). For this signal, that is, the SWEs, we do find a linear relation between not only the number of SWEs but also the amplitude of the local event to the amplitude of the BOLD signal. This particular SWE—BOLD relation may pave the way for quantitative analyses in central nervous system (CNS) disorders, in which the propagation and the recruitment of these events are perturbed.

### SWE Centered Analysis of Human Neuroimaging Data: Is it a New Translational Tool for Capturing Early Network Dysregulations in Neurodegeneration?

SWEs constitute a synchronized transition of a local population of neurons from the hyperpolarized Down state to the depolarized Up state. Indeed, most excitatory and inhibitory neurons fire exclusively during Up state (Steriade et al. 1993; Sanchez-Vives and McCormick 2000). The resulting increase in action potential rate during the SWE can be captured by single cell recordings, optical population recordings, or by EEG. These SWEs are not stationary but propagate from a local origin—like a stone thrown into a quiet lake—and recruit also distant brain regions. But this can only occur if the neurons at the propagation front can smoothly transition to the Up state. To relate back to the metaphor of throwing a stone into a lake, on a windy day with various spontaneous waves in between, a widespread propagation of the single event will not be possible. And indeed, this seems to be the case in early stages of neurodegeneration, in which local hyperexcitability occurs: The propagation of SWEs is highly distorted (Busche et al. 2015). The notion of an early hyperactivity across disease gained momentum in the recent years, from diseases as distinct as multiple sclerosis (Ellwardt et al. 2018) to Alzheimer’s disease (AD) (Busche et al. 2015) and Huntington’s disease (Arnoux et al. 2018). These early hyperactivities might contribute to the impairment of memory consolidation, via the distortion of SWE propagation as observed in the AD disease model in mice (Busche et al. 2015). Based on these observations (Busche et al. 2015), the framework put forward in this study might enable generating a biomarker for (early) network changes in human neuropsychiatric disorders.

Focusing on single-subject analyses is an effective way to assess the unique functional fingerprint of early network dysregulations, which significantly varies between individuals and cannot be preserved in group analysis. Using, for example, cross-correlations of distant brain areas (Busche et al. 2015) in relation to functional coupling analyses of SWEs could constitute a functionally significant, secondary biomarker of early network alterations way before classical neuroimaging markers of functional and structural change may be detectable (Busche et al. 2015). Hence, the logical next step is to develop further indices of SWE propagation in human imaging data and test their validity to detect hampered SWE propagation and to test their predictive value concerning the development of future disease.

## Supplementary Material

Supplementary_Figure_1_bhab516

Supplementary_Figure_2_bhab516

Supplementary_Figure_3_bhab516

Supplementary_Figure_4_bhab516

Supplementary_Figure_5_bhab516

Supplementary_Figure_6_bhab516

Supplementary_Figure_7_bhab516

Supplementary_Figure_8_bhab516

Supplementary_Figure_9_bhab516

Supplementary_Table_1_bhab516

Supplementary_Figure_Legends_bhab516
