# Supplementary figures and images for: Individual slow wave events give rise to macroscopic fMRI signatures and drive the strength of the BOLD signal in human resting-state EEG-fMRI recordings
Source: Cereb Cortex. 2022 Jan 30;32(21):4782–96. doi: 10.1093/cercor/bhab516 (PMC9627041; doi:10.1093/cercor/bhab516)

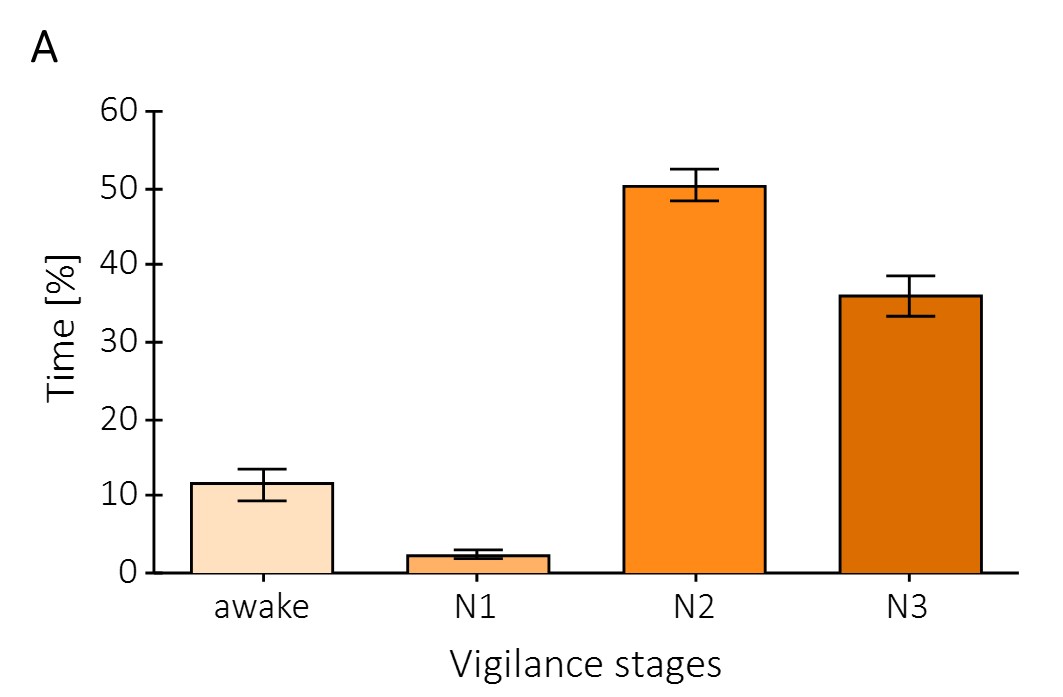

Supplement: Supplementary_Figure_1_bhab516 [file supplementary_figure_1_bhab516.jpeg]

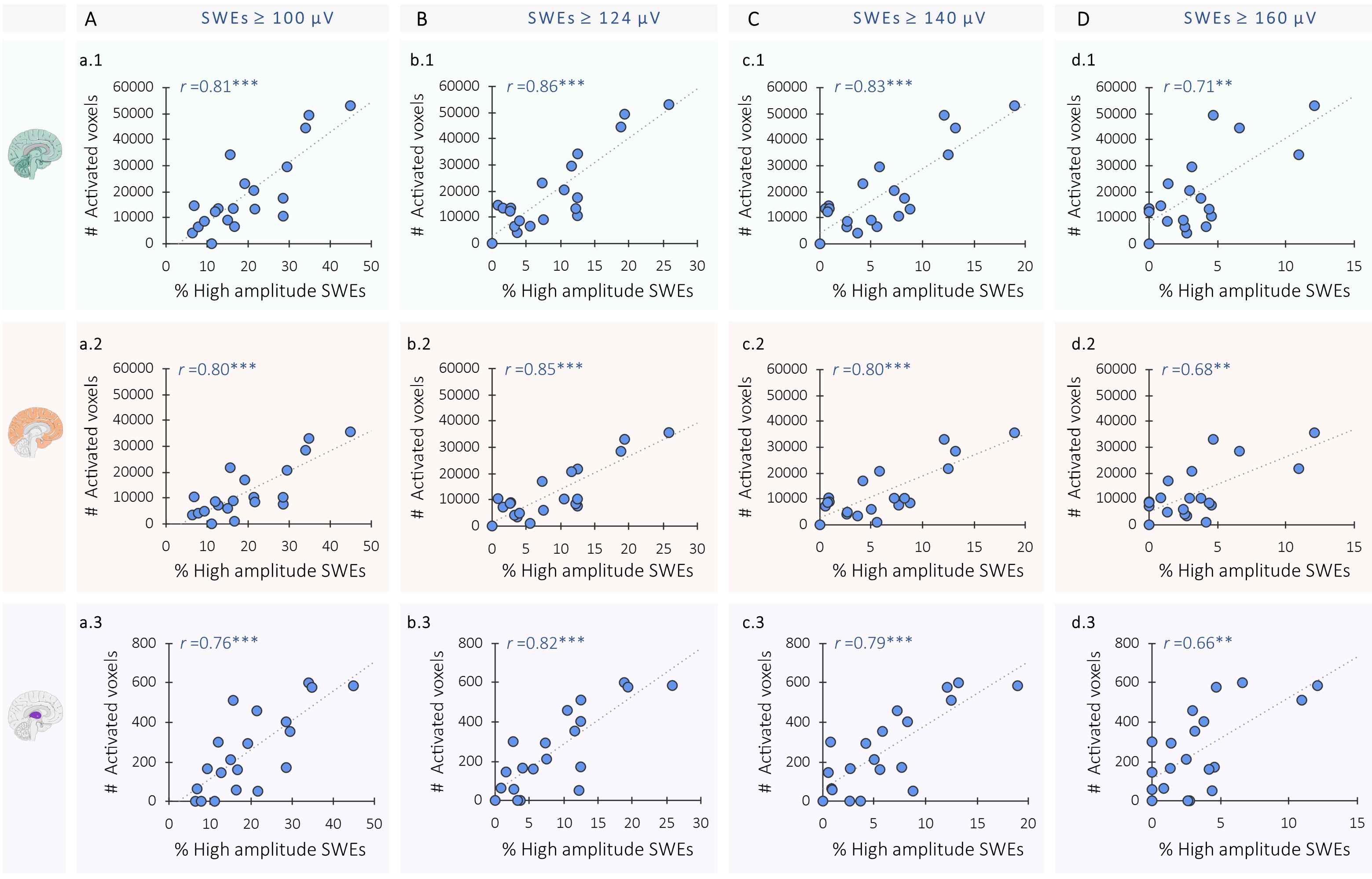

Supplement: Supplementary_Figure_2_bhab516 [file supplementary_figure_2_bhab516.jpeg]

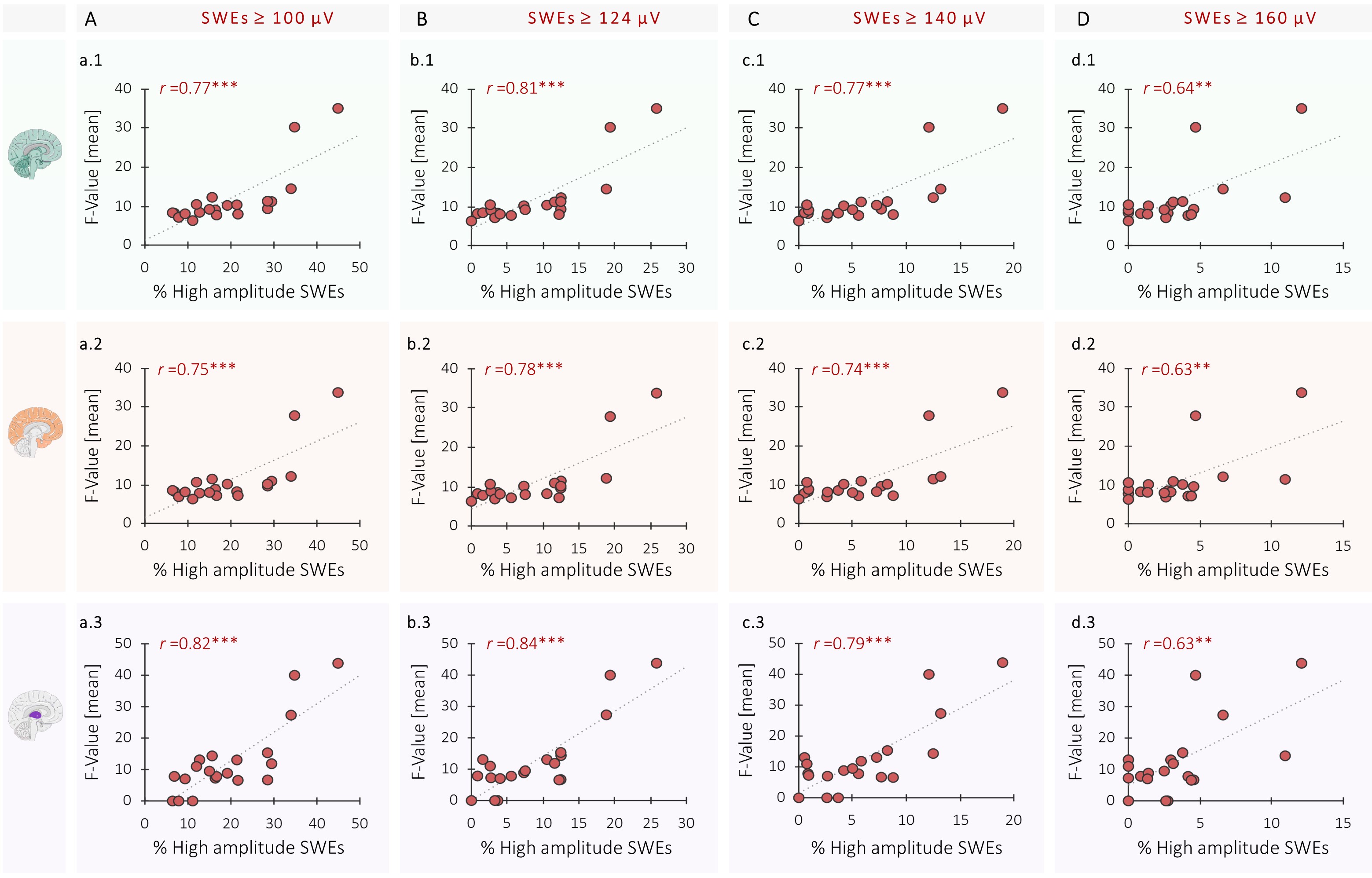

Supplement: Supplementary_Figure_3_bhab516 [file supplementary_figure_3_bhab516.jpeg]

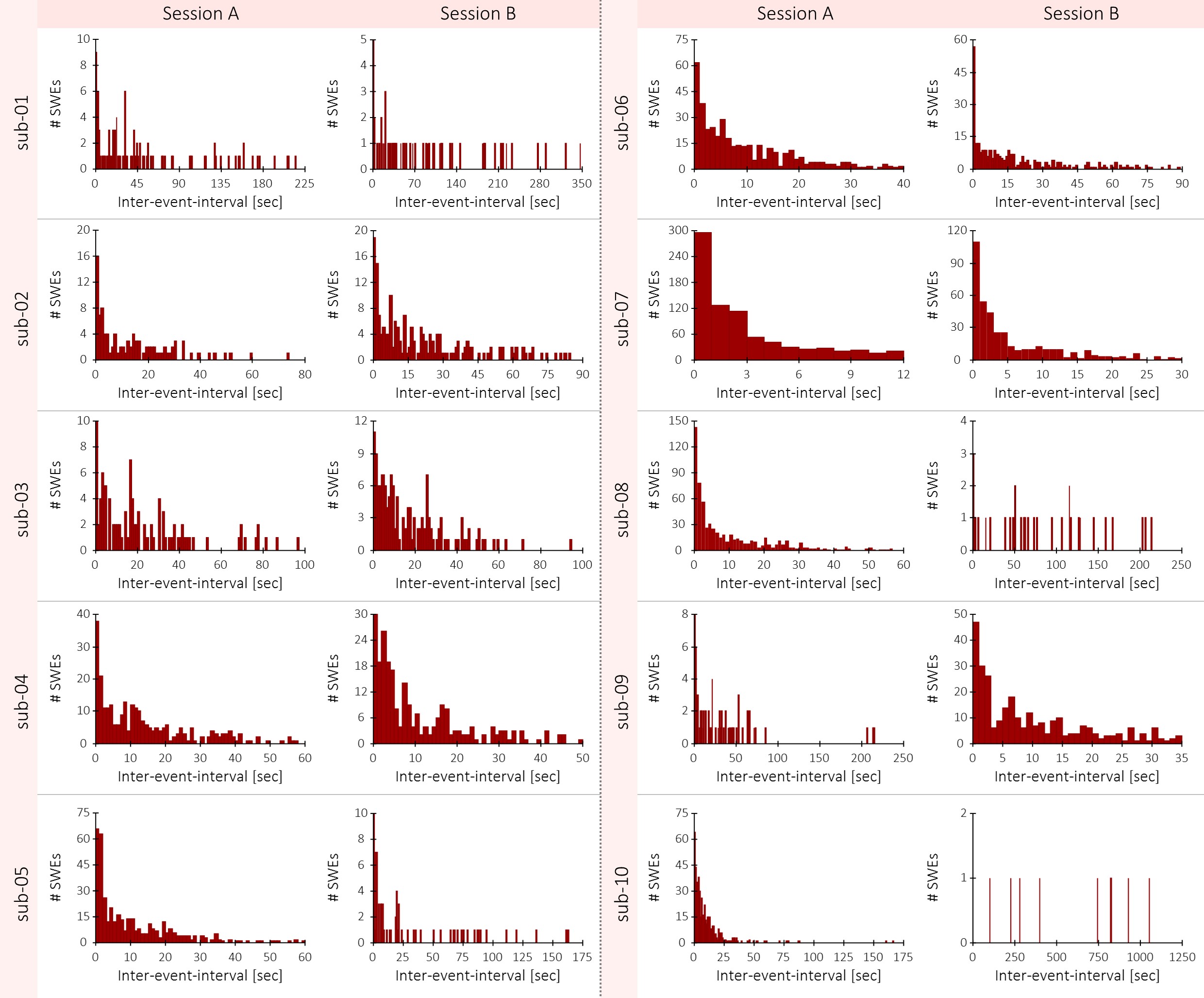

Supplement: Supplementary_Figure_4_bhab516 [file supplementary_figure_4_bhab516.jpeg]

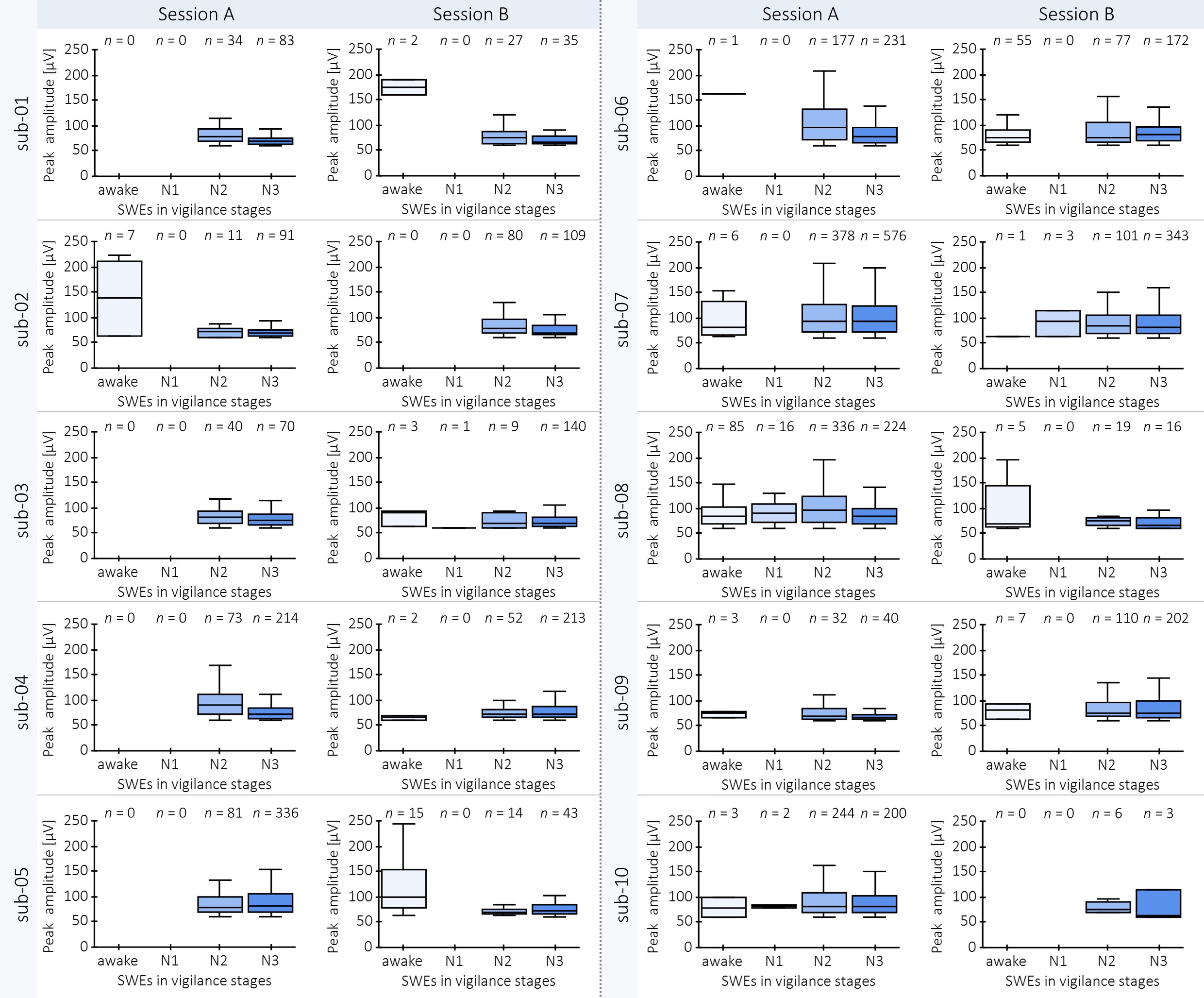

Supplement: Supplementary_Figure_5_bhab516 [file supplementary_figure_5_bhab516.jpeg]

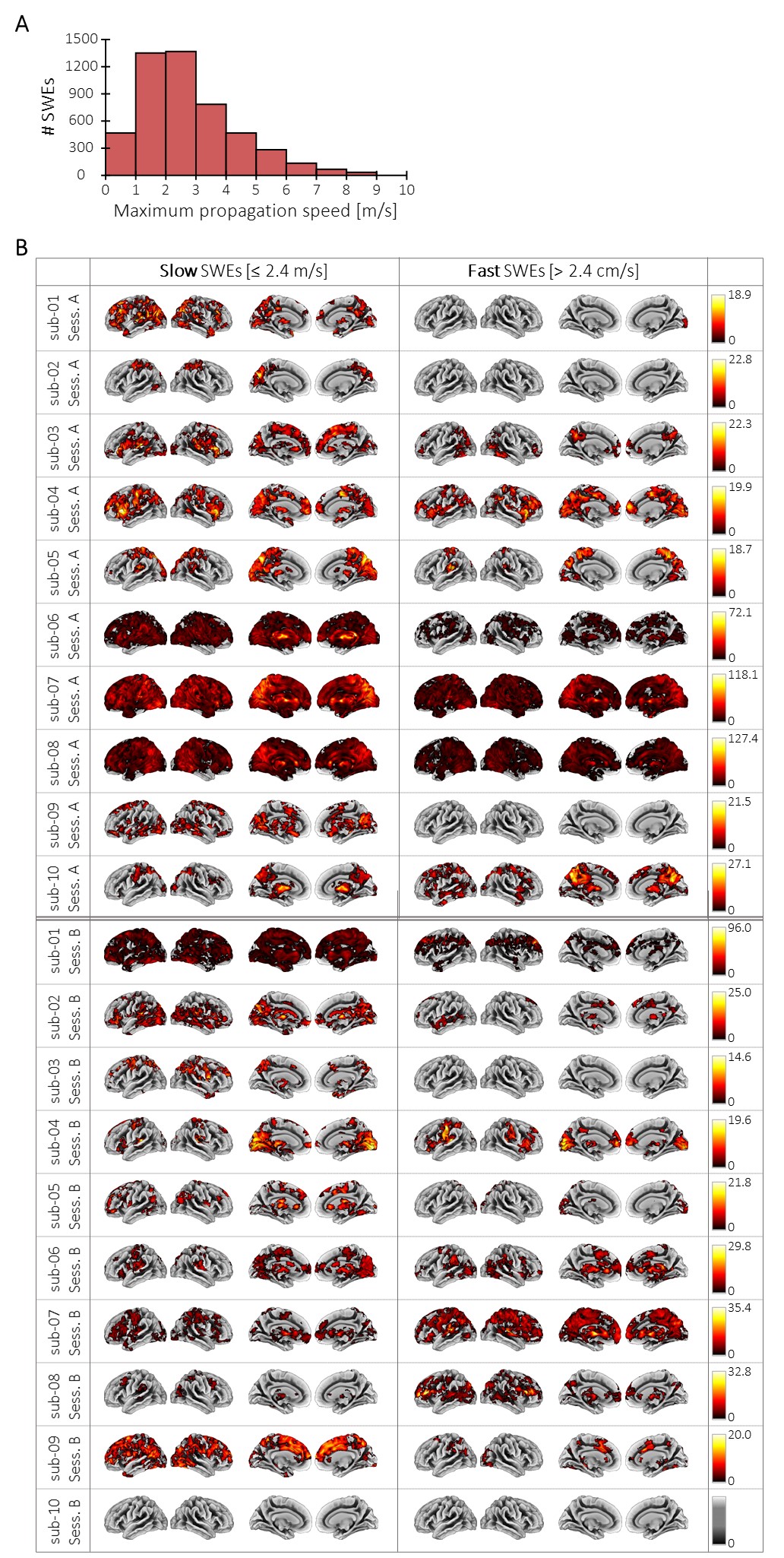

Supplement: Supplementary_Figure_6_bhab516 [file supplementary_figure_6_bhab516.jpeg]

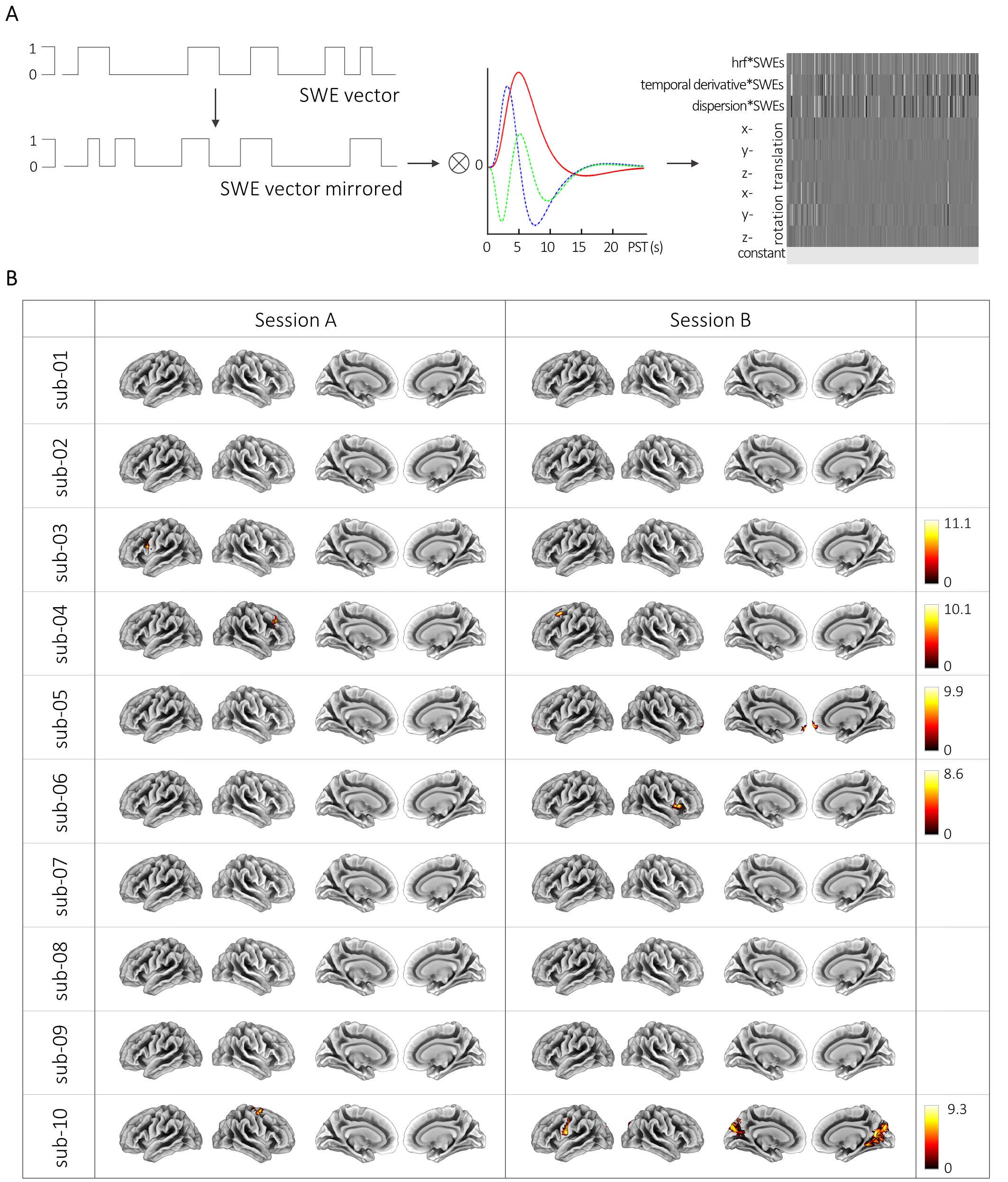

Supplement: Supplementary_Figure_7_bhab516 [file supplementary_figure_7_bhab516.jpeg]

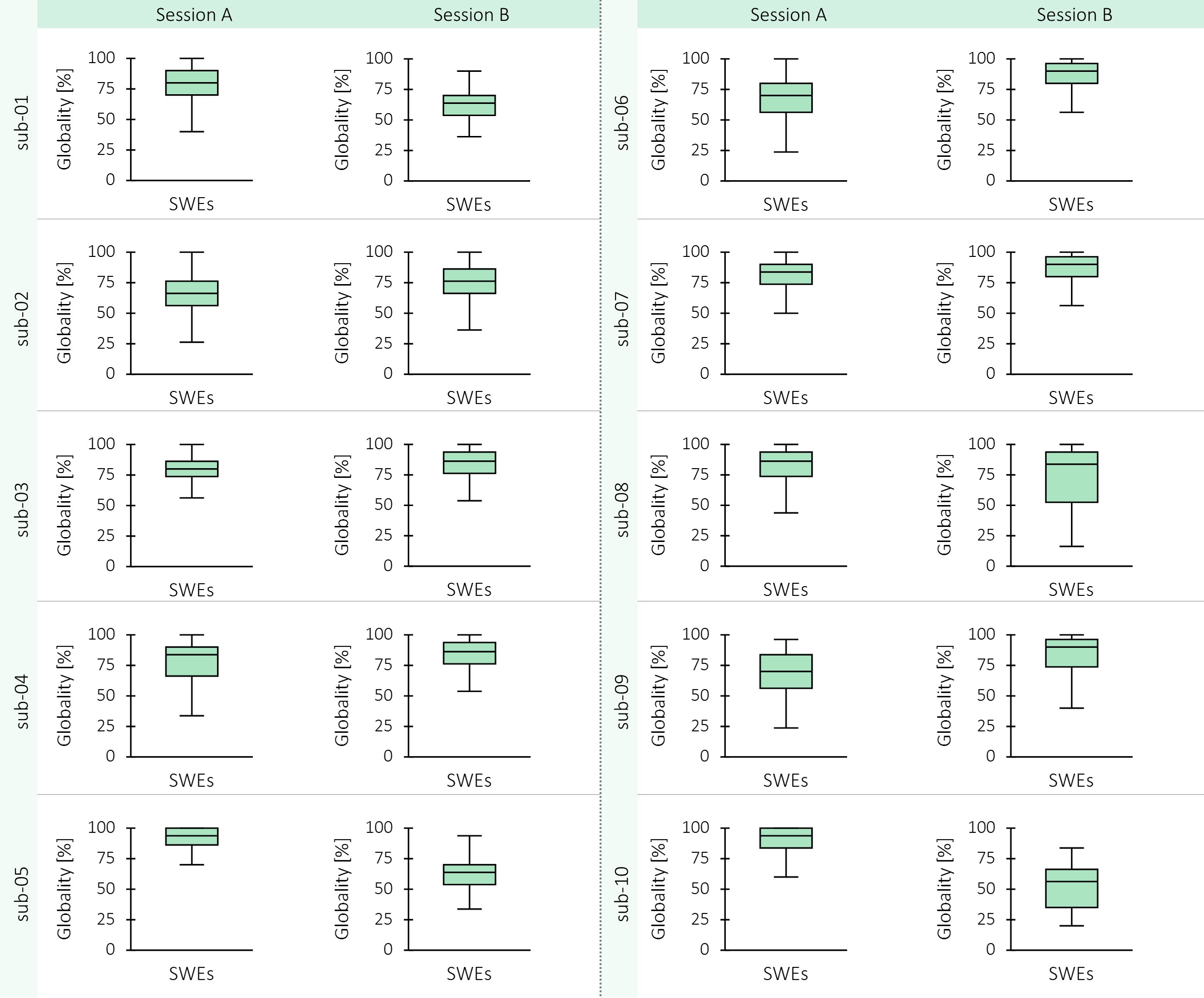

Supplement: Supplementary_Figure_8_bhab516 [file supplementary_figure_8_bhab516.jpeg]

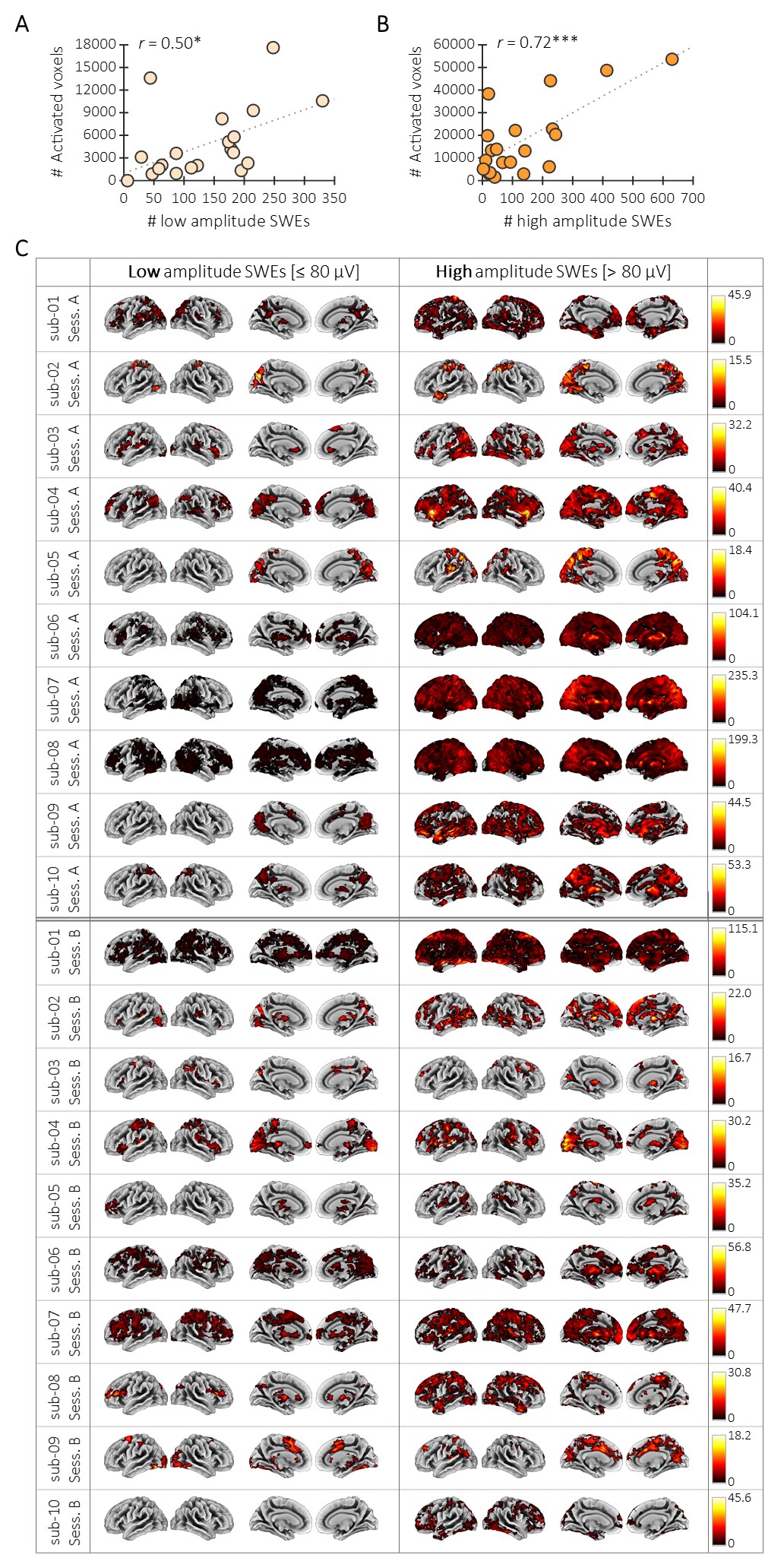

Supplement: Supplementary_Figure_9_bhab516 [file supplementary_figure_9_bhab516.jpeg]
