# Supplementary material for: Individual slow wave events give rise to macroscopic fMRI signatures and drive the strength of the BOLD signal in human resting-state EEG-fMRI recordings
Source: Cereb Cortex. 2022 Jan 30;32(21):4782–96. doi: 10.1093/cercor/bhab516 (PMC9627041; doi:10.1093/cercor/bhab516)
Supplement: Supplementary_Table_1_bhab516 [file supplementary_table_1_bhab516.docx]

**Supplementary Table 1**

Correlation Matrix

| Variable | 1 | 2 | 3 | 4 | 5 | 6 | 7 | 8 | 9 | 10 | 11 |
| --- | --- | --- | --- | --- | --- | --- | --- | --- | --- | --- | --- |
|  |  |  |  |  |  |  |  |  |  |  |  |
| 1. # Activated voxels total brain | - |  |  |  |  |  |  |  |  |  |  |
| 2. # Activated voxels cortex | 0.992*** | - |  |  |  |  |  |  |  |  |  |
| 3. # Activated voxels thalamus | 0.886*** | 0.849*** | - |  |  |  |  |  |  |  |  |
| 4. F-Value [mean] total brain | 0.856*** | 0.859*** | 0.715*** | - |  |  |  |  |  |  |  |
| 5. F-Value [mean] cortex | 0.828*** | 0.839*** | 0.670** | 0.995*** | - |  |  |  |  |  |  |
| 6. F-Value [mean] thalamus | 0.922*** | 0.901*** | 0.842*** | 0.942*** | 0.915*** | - |  |  |  |  |  |
| 7. # SWEs | 0.778*** | 0.786*** | 0.672** | 0.854*** | 0.850*** | 0.831*** | - |  |  |  |  |
| 8. Wave density (SWEs/minute) | 0.710*** | 0.722*** | 0.615** | 0.734*** | 0.731*** | 0.737*** | 0.964*** | - |  |  |  |
| 9. Mean peak amplitude [μV] | 0.830*** | 0.808*** | 0.783*** | 0.788*** | 0.761*** | 0.826*** | 0.873*** | 0.850*** | - |  |  |
| 10. Mean peak-to-peak amplitude [μV] | 0.631** | 0.624** | 0.616** | 0.646** | 0.634** | 0.638** | 0.834*** | 0.806*** | 0.824*** | - |  |
| 11. % High amplitude SWEs | 0.863*** | 0.846*** | 0.815*** | 0.809*** | 0.777*** | 0.842*** | 0.851*** | 0.827*** | 0.959*** | 0.838*** | - |

Note: Matrix shows correlations between variables obtained from SWE-related fMRI analyses such as number of activated voxels and mean F-values in different brain regions, several variables indicating the amplitude of SWEs and the number of detected SWEs.*** indicates p < 0.001. ** indicates p < 0.005.
