# Supplementary material for: Individual slow wave events give rise to macroscopic fMRI signatures and drive the strength of the BOLD signal in human resting-state EEG-fMRI recordings
Source: Cereb Cortex. 2022 Jan 30;32(21):4782–96. doi: 10.1093/cercor/bhab516 (PMC9627041; doi:10.1093/cercor/bhab516)
Supplement: Supplementary_Figure_Legends_bhab516 [file supplementary_figure_legends_bhab516.docx]

**Supplementary figure legends**

**Supplementary Figure 1.** Overall sleep architecture of the total sample. (A) Bar plot depicts the average percentage of recording time spent in wakefulness, sleep stage N1, N2 and N3 throughout the total EEG-fMRI recordings for the whole sample.

**Supplementary Figure 2.** High amplitude SWEs drive the spatial extent of the BOLD signal. Graphs show the correlations between the percentage of high amplitude SWEs detected in an individual recording session (% high amplitude SWEs) and the number of activated voxels upon SWE occurrence in the BOLD signal. For the classification of high amplitude SWEs we applied several thresholds as can be seen in panels A (≥ 100 μV), B (≥ 124 μV), C (≥ 140 μV), D (≥ 160 μV). These threshold variations served to examine the consistency of the correlation between high amplitude SWEs and the number of activated voxels upon SWE appearance. The three segments of the figure separated by color blocks show the correlations on total brain level (a.1, b.1, c.1, d.1), on cortical level (a.2, b.2, c.2, d.2) and thalamic level (a.3, b.3, c.3, d.3), respectively. *** The correlations are significant at *p* < 0.001. ** The correlations are significant at *p* < 0.01.

**Supplementary Figure 3.** High amplitude SWEs drive the amplitude of the BOLD signal. Graphs show the correlations between the percentage of high amplitude SWEs detected in an individual session (% high amplitude SWEs) and the mean F-values of SWE-related BOLD signal changes. For the classification of high amplitude SWEs we applied several thresholds as can be seen in panels A (≥ 100 μV), B (≥ 124 μV), C (≥ 140 μV), D (≥ 160 μV). These threshold variations served to examine the consistency of the correlation between high amplitude SWEs and the number of activated voxels upon SWE appearance. The three segments of the figure separated by color blocks show the correlations on total brain level (a.1, b.1, c.1, d.1), on cortical level (a.2, b.2, c.2, d.2) and thalamic level (a.3, b.3, c.3, d.3), respectively. *** The correlations are significant at *p* < 0.001. ** The correlations are significant at *p* < 0.01.

**Supplementary Figure 4**. Distribution of inter-event-intervals (IEIs) on single subject single session level. Histograms depicting the frequency distribution of IEIs show that a substantial fraction of IEIs exceed the temporal resolution of the peak of the BOLD response (~ 5- 6 seconds) (Glover 2011). These histograms were generated for both recording sessions (Session A and Session B) of each single subject (sub-01-10).

**Supplementary Figure 5**. Distribution of peak amplitudes of SWEs occurring in different vigilance stages on single subject single session level. Box-Whisker-Plots illustrate the distribution of peak amplitudes (μV) of SWEs occurring in wakefulness, in sleep stage N1, in sleep stage N2, and in sleep stage N3, respectively. These Box-Whisker Plots were both recording sessions (Session A and Session B) of each single subject (sub-01-10). The digits above the plots show the number of SWEs occurring in each vigilance stage.

**Supplementary Figure 6**. Slow versus fast SWE-related BOLD signal changes on single subject single session level. (A) Histogram illustrates the overall sample distribution of the maximum propagation speed [m/s] of SWEs exhibiting travelling streams (*N* = 4933). (B) The left column of the results table shows slow SWE-related BOLD signal changes for session A and B of subjects 01-10. The right column of the results table shows fast SWE-related BOLD signal changes for session A and B of subjects 01-10. The cut-off threshold was determined by a median split of all SWE travelling speeds [m/s]. Color bars indicate F-values.

**Supplementary Figure 7**. BOLD responses are specific to SWE occurrence. (A) Scheme of control analysis procedure. From left to right: Mirroring of the original SWE vector in time. To test the specificity of the SWE-related BOLD responses obtained in this study event vectors having the same number of events, the same distribution of event durations, and inter-event intervals, only differing in the event onset were created. The mirrored SWE vectors were convolved with the canonical HRF and its temporal and dispersion derivative for event-related fMRI analyses. An exemplary design matrix shows the individual GLM which contains the mirrored SWE vector convolved with the canonical HRF and its derivatives in time and dispersion, six movement parameters and a constant term. (B) BOLD patterns upon the occurrence of mirrored events. The results table shows an absence of BOLD responses in 15 out of 20 recording sessions. For the remaining five sessions (sub-03-A, sub-04-B, sub-05-B, sub-06-B, and sub-10-B) there are random localized BOLD responses with F-values substantially differing from the responses obtained by the original SWE vectors.

**Supplementary Figure 8**. Distribution of SWE globality (percentage of channels involved in each SWE) on single subject single session level. Box-Whisker-Plots illustrate the distribution SWE globality [%] for both recording sessions (Session A and Session B) of each single subject (sub-01-10).

**Supplementary Figure 9**. Low amplitude versus high amplitude SWE-related BOLD signal changes on single subject single session level. (A) Scatter plot shows the correlation between the number of low amplitude SWEs and the number of activated voxels upon low amplitude SWEs occurrence. (B) The correlation between the number of high amplitude SWEs and the number of activated voxels upon high amplitude SWEs occurrence displayed in a scatter plot. (C) The left column of the results table shows low amplitude SWE-related BOLD signal changes for session A and B of subjects 01-10. The right column of the results table shows high amplitude SWE-related BOLD signal changes for session A and B of subjects 01-10. The cut-off threshold was determined by a median split of the peak amplitudes of all SWEs detected in this study (*N* = 5459). Color bars indicate F-values. *** The correlation is significant at *p* < 0.001. * The correlation is significant at *p* < 0.05.
